# Mind the mechanical strength: tailoring a 3D matrix to encapsulate isolated human preantral follicles

**DOI:** 10.1093/hropen/hoad004

**Published:** 2023-02-17

**Authors:** Arezoo Dadashzadeh, Saeid Moghassemi, Alexis Peaucelle, Carolina M Lucci, Christiani A Amorim

**Affiliations:** Pôle de Recherche en Physiopathologie de la Reproduction, Institut de Recherche Expérimentale et Clinique, Université Catholique de Louvain, Brussels, Belgium; Pôle de Recherche en Physiopathologie de la Reproduction, Institut de Recherche Expérimentale et Clinique, Université Catholique de Louvain, Brussels, Belgium; Institut Jean-Pierre Bourgin, INRAE, AgroParisTech, Université Paris-Saclay, Versailles, France; Department of Physiological Sciences, Institute of Biological Sciences, University of Brasília, Brasília, Brazil; Pôle de Recherche en Physiopathologie de la Reproduction, Institut de Recherche Expérimentale et Clinique, Université Catholique de Louvain, Brussels, Belgium

**Keywords:** ovary tissue engineering, PEGylated fibrin, hydrogel, response surface methodology, mechanical properties, human follicle growth, polyethylene glycol

## Abstract

**STUDY QUESTION:**

Would a hydrogel with similar mechanical properties to the human ovarian cortex support preantral follicle development?

**SUMMARY ANSWER:**

Yes, our tailored PEGylated fibrin hydrogel was shown to significantly improve follicle growth *in vitro.*

**WHAT IS KNOWN ALREADY:**

One of the main challenges in developing an engineered ovary is to provide a 3D matrix that supports the follicle architecture and the interaction between granulosa cells and the oocyte as they are essential for folliculogenesis. Thanks to its biocompatibility and bioactivity, fibrin has been employed to fabricate a 3D matrix to encapsulate ovarian follicles. However, follicles lose their physical support within a few days owing to rapid fibrin degradation. Therefore, different strategies, including physical and chemical modifications, have been developed to enhance the stability of fibrin.

**STUDY DESIGN, SIZE, DURATION:**

By developing a matrix made of a synthetic (polyethylene glycol: PEG) and natural polymer (fibrin), we aimed to overcome fibrin degradation by the chemical reaction of PEGylation and tailor a PEGylated fibrin hydrogel formulation with mechanical strength similar to the ovarian cortex in women of reproductive age. To this end, response surface methodology was employed to obtain a tailored formulation of PEGylated fibrin. This hydrogel was then tested to encapsulate and support isolated human preantral follicles *in vitro.*

**PARTICIPANTS/MATERIALS, SETTING, METHODS:**

A PEGylated fibrin formulation was tailored using mathematical modeling software to mimic the mechanical properties of human ovarian tissue at reproductive age. Human preantral follicles were isolated from 11 patients of reproductive age and encapsulated in the tailored hydrogels, which were cultured *in vitro* for 4 or 7 days. Follicle survival and diameter were assessed on Days 1 and 7. Furthermore, the follicles were subjected to confocal microscopy to evaluate their growth (Ki67 staining) on Day 7 and analyze cell–cell communication (connexin 43 and transzonal projection staining) on Day 4.

**MAIN RESULTS AND THE ROLE OF CHANCE:**

In this study, mathematical modeling was applied to achieve the biomechanically tailored PEGylated fibrin formulation by targeting the specific goal of 3178 ± 245 Pascal, Young’s modulus of ovarian cortical tissue in reproductive-age women. Our results demonstrated that the PEGylated fibrin hydrogel consisting of 39.06 mg/ml of PEGylated fibrinogen and 50.36 IU/ml of thrombin was the optimum condition with the desirability of 97.5%. This tailored hydrogel yielded a high follicle survival rate (83%) after 7 days of *in vitro* culture and supported its development up to the secondary stage. Follicle growth was confirmed by the presence of Ki67-positive granulosa cells on Day 7. Additionally, connexin 43 and Phalloidin staining indicated the retention of connections between granulosa cells and the oocyte.

**LARGE SCALE DATA:**

N/A.

**LIMITATIONS, REASONS FOR CAUTION:**

In this study, our tailored hydrogel was only tested *in vitro*, which is not the same as the physiological environment. It is crucial to conduct a study assessing the follicles following their encapsulation in the tailored hydrogel and transplantation, which will be the next step of our investigation.

**WIDER IMPLICATIONS OF THE FINDINGS:**

The findings from this study introduced a suitable biomaterial similar to the ovarian cortex in reproductive-age women in terms of biomechanical properties for encapsulating human preantral follicles. This biomaterial allowed the radial growth of follicles and preserved their viability. Furthermore, PEGylation improved the stability of fibrin and the physical support of follicles.

**STUDY FUNDING/COMPETING INTEREST(S):**

This study was supported by grants from the Fondation Louvain (PhD scholarship awarded to S.M., as part of a legacy from Mr Frans Heyes, and PhD scholarship awarded to A.D. as part of a legacy from Mrs Ilse Schirmer). The authors declare no competing interests.

WHAT DOES THIS MEAN FOR PATIENTS?

Survival rates of leukemia patients have been steadily increasing but, owing to cancer treatment, some survivors will face infertility at a very young age. One of the strategies under development to restore fertility in these patients is the transplantable ‘engineered ovary’, which should mimic the normal ovary as closely as possible. To assemble the engineered ovary, it is necessary to encase isolated preantral follicles (follicles at an early stage of growth that contain one immature egg) and ovarian cells in a 3D matrix. Developing this matrix is one of the main challenges in creating an engineered ovary, as it should support the follicle architecture as well as the interaction between granulosa cells (which produce estrogen and progesterone) and the oocyte (egg), as they are essential for follicle development. This study demonstrates that a matrix made of a synthetic (polyethylene glycol) and natural polymer (fibrin), with mechanical strength similar to that of the outer layer of the ovary in women of reproductive age, can successfully be used to encapsulate human preantral follicles. Indeed, we have shown that our 3D matrix yielded a high follicle survival rate and supported follicle growth to the secondary stage.

## Introduction

While cryopreservation and transplantation is one of the strategies for preserving fertility in cancer patients, it is not advised for cancer patients with tumors at high risk of metastasis to the ovary (Amorim and Shikanov, 2016; Dolmans and Amorim, 2019; Jafari *et al.*, 2021). An engineered ovary, including follicles as functional units, can be a promising strategy for maintaining fertility in these patients. In this approach, follicles are encapsulated in a 3D matrix, which should be biocompatible, biodegradable, easy to handle, and able to deliver oxygen and nutrients and protect the spatial follicle morphology (Dadashzadeh *et al.*, 2021). One of the most promising candidates for follicle encapsulation is fibrin (Luyckx *et al.*, 2013, 2014; Chiti *et al.*, 2016, 2017a,b, 2018a, b; Manavella *et al.*, 2018). Nevertheless, the fast fibrin degradation causes follicles to lose their physical support in a few days (Ahmed *et al.*, 2008; Galler *et al.*, 2011; Benavides *et al.*, 2015; Amorim, 2016), which negatively affects their survival. Chemical modification can be an alternative to overcome this limitation (Zhang *et al.*, 2006; Shapira-Schweitzer and Seliktar, 2007; Shpichka *et al.*, 2018). For instance, PEGylated fibrin hydrogels have shown bioactivity similar to native fibrin with the added advantage of controllable physical properties and biodegradation (Mironi-Harpaz *et al.*, 2014). Indeed, polyethylene glycol (PEG) molecules serve as protease shields to decrease protein proteolysis, thanks to their large hydrodynamic radius (Lawrence and Price, 2016). As a preliminary study of PEGylated fibrin for ovary tissue engineering, our group demonstrated that PEGylation increases the stability of fibrin matrices and supports the viability and proliferation of ovarian isolated stromal cells (Dadashzadeh *et al.*, 2021).

Ideally, the engineered ovary should mimic the normal ovary as closely as possible to allow preantral follicles to survive and develop. One of the necessary cues to mimic is the mechanical signal in the natural ovarian cortex. When follicles grow, the compressive pressure applied from surrounding biomaterials affects their development and diameter expansion (Smith *et al.*, 2010; Chiti *et al.*, 2018a,b). Thus, mimicking the ovary’s mechanical properties is critical when designing an engineered ovary as it will allow radial follicle growth (Shikanov *et al.*, 2009; Smith *et al.*, 2010; Choi *et al.*, 2014; Kim *et al.*, 2020). In this study, therefore, we aimed to tailor our PEGylated fibrin hydrogel considering the mechanical properties of the human ovarian cortex at reproductive age. To this end, we have chosen Young’s modulus of ovarian cortex at reproductive age (Ouni *et al.*, 2021) as a reference to prepare our hydrogel with similar mechanical characteristics. Then, the human preantral follicles were isolated and encapsulated in this mechanically designed PEGylated fibrin hydrogel to assess its ability to preserve the 3D structure of follicles and provide an appropriate matrix for their growth.

## Materials and methods

### Ethics

The use of the human ovarian cortex was approved by the Institutional Review Board of the Université Catholique de Louvain on 13 May 2019 and 25 May 2019 (IRB references 2012/23MAR/125 and 2018/19DEC/475).

### Materials and reagents

Human fibrinogen (F3879), human thrombin (T; T7009), O,O′-Bis[2-(N-Succinimidyl-succinylamino)ethyl]polyethylene glycol (NHS-PEG-NHS; 2000 Da; 713783), CaCl_2_ (C5080), sodium pyruvate solution (s8636), Liberase DH (5401054001), and DNAse I (10104159001) were purchased from Sigma-Aldrich Chemical Co. (Darmstadt, Germany).

Dulbecco’s modified Eagle’s medium F-12 nutrient mixture (DMEM/F12; 21041-025), minimum essential medium (MEM; 42360-024), heat-inactivated fetal bovine serum (HI FBS; 16140-071), Insulin-Transferrin-Selenium (ITS; 41400-045), Dulbecco’s phosphate buffered saline (DPBS) containing calcium and magnesium (14040091), and DPBS without calcium and magnesium (14190144) were obtained from Thermo Fisher (Paisley, UK). Antibiotic and antimycotic (Anti-Anti; A5955) was obtained from Thermo Fisher (Green Island, NY, USA). All other chemicals were of the highest grade commercially available.

### Experimental design

This study was divided into three experiments: synthesis of a tailored PEGylated fibrin hydrogel; evaluation of the hydrogel suitability for an engineered ovary by encapsulation of human preantral follicles and *in vitro* study; and assessment of the hydrogel capability to support cell migration.

First, mathematical modeling was applied to discover a PEGylated fibrin formulation with a similar mechanical strength to ovaries at reproductive age. Then, the tailored PEGylated fibrin formulation was used to encapsulate human preantral follicles. The effect of the hydrogel encapsulation on follicle survival and development was assessed *in vitro* by morphological assessment of follicles and measuring their diameters, as well as Ki67, connexin 43, and transzonal projections (TZP) immunostaining. Moreover, the impact of the hydrogel on supporting cell migration was evaluated by using ovarian stromal cells encapsulated in fibrin clots, which were localized in the tailored PEGylated fibrin.

### Tailoring the PEGylated fibrin hydrogel

#### Establishing the biomechanical properties of the hydrogel

In this study, Design-Expert^®^ software (Version 12.0, State-Ease Minneapolis, MN, USA) was employed to tailor the hydrogel formulation with matching biomechanical properties to ovarian tissue in women at reproductive age (Ouni *et al.*, 2021). Response surface methodology (RSM)-based central composite design (CCD) was used to acquire a proper mathematical model that can significantly predict and visualize the effect of the independent variables on the mechanical results of PEGylated fibrin hydrogels in the experimental conditions. Here, PEG:Fib (10:1) and thrombin concentration were chosen as independent variables, and their Young’s modulus was selected as a dependent variable. According to the software, 21 runs were designed to obtain the best-fit models. All the numerical independent and dependent variables and their ranges are summarized in Table I.

**Table I:** Numerical dependent (Young’s modulus) and independent variables (PEG: Fib (10:1) and thrombin) with their ranges introduced to Design-Expert*^®^* software.

				Coded value			
Experimental parameters	Name	Unit	Type	Low (−1)	Medium (0)		High (+1)
X_1_	PF	mg/ml	Numeric	15	45		75
X_2_	T	IU/ml	Numeric	20	50		80

Response	Name	Unit	Type	Min		Max	
Y_1_	Young’s modulus	Pa	Numeric	1235.29 ± 128.27		15871.75 ± 1960.25	

PF, PEGylated fibrinogen; T, thrombin; Pa, Pascal.

#### Preparation of PEGylated fibrin hydrogels

To test the mechanical properties of 21 designed formulations (Table II), human fibrinogen was solubilized in DPBS at a concentration of 85 mg/ml. After 2 h incubation at 37°C, the solution was filtered using a 0.20-mm syringe filter. NHS-PEG-NHS solution in DPBS was prepared at 25, 17.48, and 5 mg/ml and syringe filtered. NHS-PEG-NHS solutions were added to the fibrinogen in different volume ratios to create a 10:1 M ratio of PEGylated fibrinogen (PEG:Fib 10:1 (PF)) with different concentrations of 75, 70, 45, 20, and 15 mg/ml (PF75, PF70, PF45, PF20, and PF15, respectively), mixed thoroughly, and incubated at 37°C. Human thrombin was reconstituted and diluted in 40 mM CaCl_2_ to achieve concentrations of 80, 75, 50, 25, and 20 IU/ml (T80, T75, T50, T25, and T20, respectively) and incubated at 37°C before use. Equal volumes of PF and thrombin were mixed and incubated at 37°C to produce PEGylated fibrin hydrogels for measuring their Young’s modulus.

**Table II:** Composition of factorial formulations designed by Design-Expert*^®^* software.

Points	Replication	Coded level		Actual level of variables	
		PF	T	PF (mg/ml)	T (IU/ml)
(−1,0)	2	−1	0	15	50
(0,−1)	2	0	−1	45	20
(0,0)	5	0	0	45	50
(0,+1)	2	0	+1	45	80
(+1,0)	2	+1	0	75	50
(−α,−α)	2	−α	−α	20	25
(−α,+α)	2	−α	+α	20	75
(+α,−α)	2	+α	−α	70	25
(+α,+α)	2	+α	+α	70	75

PF, PEGylated fibrinogen; T, thrombin.

#### Atomic force microscopy

Hydrogels were freshly prepared in a 100% humidity atmosphere on glass slides. They were incubated for 15 min at 37°C and imaged in PBS solution. The maximum indentation force was set from 10 to 60 nN depending on the gel composition to archive a maximum indentation of more than 500 nm and no more than 1 µm. The cantilever used was ‘Novascan’ (Nanosensors Headquarters, Neuchâtel, Switzerland) SD-Sphere-NCH-S tips with a nominative spring constant of 0.56 N/m; the one used was estimated through the thermo-noise method to be 0.295 N/m with silicon point probe tips of a 2 µm radius.

All force spectroscopy experiments were performed on a JPK nanowisared 1 AFM (JPK Instruments AG, Berlin, Germany) as previously described (Ouni *et al.*, 2021). Briefly, the elastic modulus of hydrogels was determined as follows: an atomic force microscopy (AFM) cantilever loaded with a spherical tip was used to indent the sample over a 100 × 100 μm square area, and within the area 8 × 8 measurements were made, resulting in 48 force indentation experiments; each force-indentation experiment was treated with a Hertzian indentation model to extract Young’s modulus (E_A_). The speed of the probe was set to 5 µm s^−1^. The E_A_ was calculated using the JPK Data Processing software (ver. Spm - 4.0.23, JPK Instruments AG, Germany), which allows for a standardized analysis using a standard Hertzian contact model (Ouni *et al.*, 2021). Only the retraction curve was used in our analyses, as is typically the case in nano-indentation experiments. A Poisson ratio of 0.5 was assumed for the material.

#### Scanning electron microscopy

Hydrogels were fixed in 4% paraformaldehyde overnight, washed in 0.1 M cacodylate buffer, permeated with glycerol (15% for 30 min and 30% overnight), plunged into liquid nitrogen, and freeze-fractured using a blade. Samples were dehydrated by a graded series of acetone (50%, 70%, 80%, 90%, and three times 100%) at 15 min intervals and subsequently placed in a critical point dryer (Balzers CPD030). Samples were coated with gold (Leica SCD500 coater) and analyzed and imaged using a scanning electron microscope (Jeol JSM 7001F).

### Testing the PEGylated fibrin hydrogel for the development of isolated preantral follicles

#### Collection of ovarian cortex biopsies

Human ovarian tissue biopsies (from 11 patients) were taken after obtaining informed consent from patients aged 18 and 35 years old who were going through gynecology-related laparoscopic surgery. The biopsies were immediately transferred to the laboratory in the MEM at 4°C to remove the medullar part, cut into small pieces (max. 5 × 5 mm^2^), and frozen following our routine procedure (Gallardo *et al.*, 2018).

#### Ovarian follicle isolation

After ovarian tissue thawing (Gallardo *et al.*, 2018), the human preantral follicles were isolated according to the Chiti *et al.* (2017a,b) protocol. Briefly, the tissue fragments were minced with the aid of a tissue chopper (McIlwain, Campden Instruments, Loughborough, UK), and then enzymatically digested with Liberase DH and DNase I for sequential 30 min periods until complete digestion was achieved. At the end of each 30 min incubation, the digested tissue suspension was filtered through a 100 μm cell strainer (43-50100-01, pluriSelect, Leipzig, Germany), and the fragments remaining in the filter transferred to a new enzymatic solution and incubated for another 30 min. The cell strainer filtrate was inactivated with an equal volume of DPBS without calcium and magnesium supplemented with 10% HI FBS, and centrifuged for 10 min (500*g*, 4°C). Then, the supernatant was removed, leaving 5 ml of the supernatant to resuspend the pellet. The preantral follicles were picked up under a stereomicroscope and picked up follicles were put in droplets of inactivated solution on the plastic Petri dishes and placed on a cold plate (4°C) until they were encapsulated in the tailored hydrogel.

#### Follicle encapsulation and *in vitro* culture

A total of 2 µl of the follicle suspension containing around 10 follicles was mixed with 13 µl PEGylated fibrinogen in a µ-slide 8-well^high^ Bioinert plate (80800; Ibidi, Gräfelfing, Germany) to achieve the PF with the calculated concentration from the RSM results. Then, the PF suspension was polymerized by adding 15 µl of the optimized thrombin concentration. The follicles were then cultured for 4 or 7 days in a culture medium containing DMEM/F12 supplemented with 10% HI FBS, 1% Anti-Anti, 1% ITS, and 2 mM sodium pyruvate at 37°C in a humidified incubator with 5% CO_2_. Half of the medium was replaced with fresh culture medium every other day.

#### Analysis of follicle survival and growth

Follicle survival was evaluated morphologically according to the Chiti *et al.* (2022) procedure. Briefly, follicles were assessed using inverted microscopy (Leica DMIL, Diegem, Belgium) on Days 1 and 7, and the ones containing extruded oocytes, oocytes not entirely covered by granulosa cells, dark granulosa cells or oocytes, or with reduced diameter were considered dead. Follicle growth was evaluated by measuring their diameter on Days 1 and 7 by averaging two perpendicular diameters measurements using the inverted microscope with a scale in the objective lens.

#### Immunofluorescence analysis

Follicle growth and cell–cell communication in follicles were evaluated by Ki67, connexin 43 (Cx43), and TZP staining on Days 4 and 7, by adapting the protocols from Ouni *et al.* (2022) and Bus *et al.* (2021). Briefly, at the end of each culture period (4 or 7 days), the hydrogels were washed with PBS. Follicles were then fixed and permeabilized with 4% paraformaldehyde and 1% Triton X-100 for 1 h at room temperature. Subsequently, they were blocked in DPBS supplemented with 0.05% Triton X-100, 5% normal goat serum, and 0.2% sodium azide overnight at 4°C with shaking. Afterward, the hydrogels were incubated in monoclonal mouse anti-human Ki67 antigen (1:100) or rabbit anti-human connexin-43 (1:268) diluted in blocking solution for 2 days at 4°C with shaking. Then, the hydrogels containing the follicles were washed three times in PBS containing 0.05% Triton X-100 at room temperature. After 1 day, the secondary antibodies were diluted in blocking solution and respectively added to the Ki67 and Cx43 staining hydrogels for 2 days at 4°C with shaking: Alexa Fluor 647 goat anti-mouse (1:250) and Alexa Fluor 488, goat anti-rabbit (1:250). As the cytoskeleton of TZPs is composed of filamentous actin (F-actin), the follicles in the Cx43 staining group were washed and incubated in Alexa Fluor 568 Phalloidin (1:400) diluted in DPBS for 1 day at 4°C with shaking. After washing hydrogels with DPBS, follicles were counterstained with DPBS–diluted Hoechst (1:100) at 4°C, overnight, to stain the nuclei, then rinsed with DPBS for 1 day at room temperature. Subsequently, 200 µl of RapiClear 1.49 solution (Sunjin Lab, Hsinchu City, Taiwan) was added at 37°C for 1 h before image acquisition by confocal microscopy (LSM800; ZEISS) equipped with four laser lines of 405, 488, 561, and 640 nm.

The images of follicles in the Ki67 and Cx43 staining groups were captured using 20×- and 40×-oil immersion objectives, respectively. The categorization of Cx43 and TZPs was performed based on the Bus *et al.* (2021) protocol, where Cx43 was classified as positive or negative in case of the presence or absence of this staining. Moreover, TZPs were considered a total absence, partial absence, or complete when no physical connection was observed between oocyte and granulosa cells, gaps existed between oocyte and granulosa cells, or no contact was lost between oocyte and granulosa cells, respectively (Bus *et al.*, 2021). Negative controls were created by omitting the Cx43-primary antibody and the TZPs staining.

### Testing the PEGylated fibrin hydrogel for the migration of ovarian cells

#### Collection of ovarian cortex biopsies

Human ovarian tissue biopsies were taken from multi-organ donor patients. The ovaries were immediately transferred to the laboratory in the MEM at 4°C to remove the medullar part, cut into small pieces (max. 5 × 5 mm^2^), and frozen following our routine procedure (Gallardo *et al.*, 2018).

To evaluate the ability of the tailored hydrogels to support cell migration, human ovarian fragments were thawed (Gallardo *et al.*, 2018) and used to isolate cells using the protocol described by our group (Moghassemi *et al.*, 2021). Briefly, the ovarian tissues were mechanically minced using a tissue chopper before being enzymatically digested at 37°C for 75 min in a solution containing Liberase DH and DNAse I. Then, by adding an equal volume of DPBS without calcium and magnesium supplemented with 10% FBS, enzymatic digestion was stopped. The suspension was then filtered through 100 and 30 µm cell strainers. Afterwards, the suspension was centrifuged for 10 min (500*g*, 4°C) and the pellet resuspended in cell culture medium containing 10% FBS, 1% Anti-Anti, and DMEM/F12, and cultured at 37°C in a humidified incubator with 5% CO_2_. The culture medium was replaced every other day and once confluence was achieved, cells were subcultured.

The test migration was based on the protocol of Lei and Segura (2009). To this end, 300 000 ovarian stromal cells at Passage 4 were encapsulated in 10 µl fibrin clots made of 4 mg/ml fibrinogen and 2 IU/ml thrombin. The fibrin/cell clots were put into the PEGylated fibrin to be incorporated within the tailored hydrogel. The fibrin/cells clot and PEGylated fibrin hydrogel were maintained i*n vitro* in a medium consisting of DMEM/F12 supplemented with 10% FBS and 1% Anti–Anti. The hydrogels were incubated at 37°C for 4 days in a humidified incubator. The outgrowth and migration of cells from fibrin clots were tracked on Days 0, 1, 2, 3, and 4 under the inverted microscope.

### Statistical analysis

The quantitative data are given as mean ± SD, one-way ANOVA was used to statistically evaluate the data, and a *P*-value of <0.05 was used to determine statistical significance. In graphs, the error bars represent one sample SD. Unpaired Student’s *t*-tests were used to compare individual conditions, with Bonferroni multiple testing corrections performed where necessary.

## Results

### Biomechanical tailoring of PEGylated fibrin hydrogels

**Figure 1.**
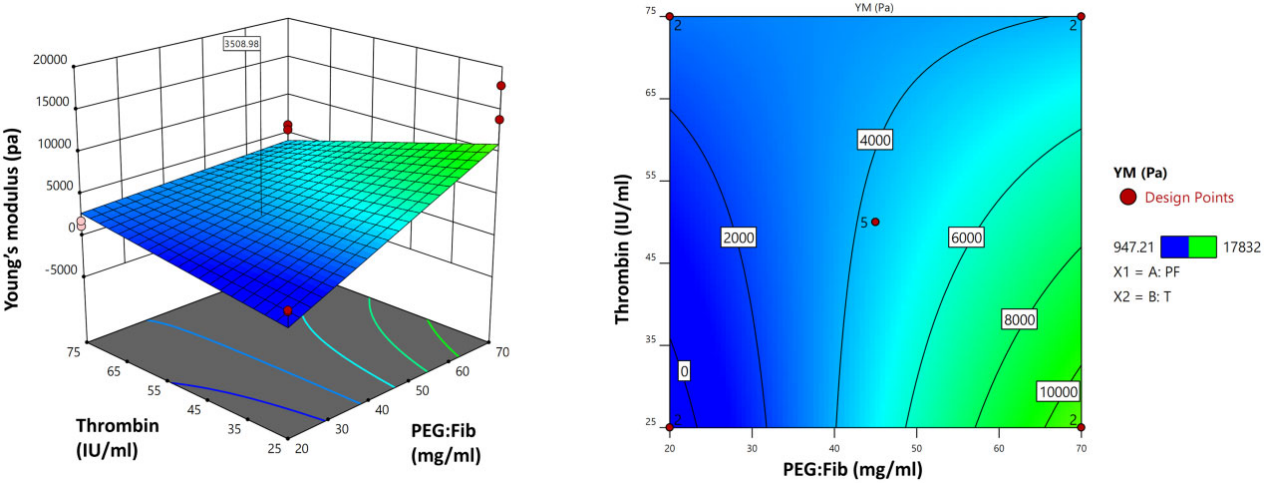
**PEGylated fibrin Young’s modulus.** Response surface methodology of PEGylated fibrin hydrogel Young’s modulus based on different concentrations of PEGylated fibrinogen and thrombin. Pa, Pascal; YM, Young’s modulus; RSM, response surface methodology; PEG, polyethylene glycol; PF, PEGylated fibrinogen; T, thrombin.

In this study, the biomechanically tailored formulation was obtained by mathematical modeling and regression analysis, which was performed to fit the data from CCD into a two-factorial interaction regression model. As a result, the following model equation was suggested for Young’s modulus of PEGylated fibrin hydrogels based on the results obtained from AFM (Fig. 1):


$$Y_{1}=4388.96+3140.69\;*\;X_{1}\;–\;982.11\;*\;X_{2}\;–\;2393.72\;*\;X_{1}X_{2};\;\left(P-\text{value}=0.0022,\;F-\text{value}=7.38\right).$$

The model *P*-value was <0.05 and the model *F*-value was 7.38, which implies the model is significant.

**Figure 2.**
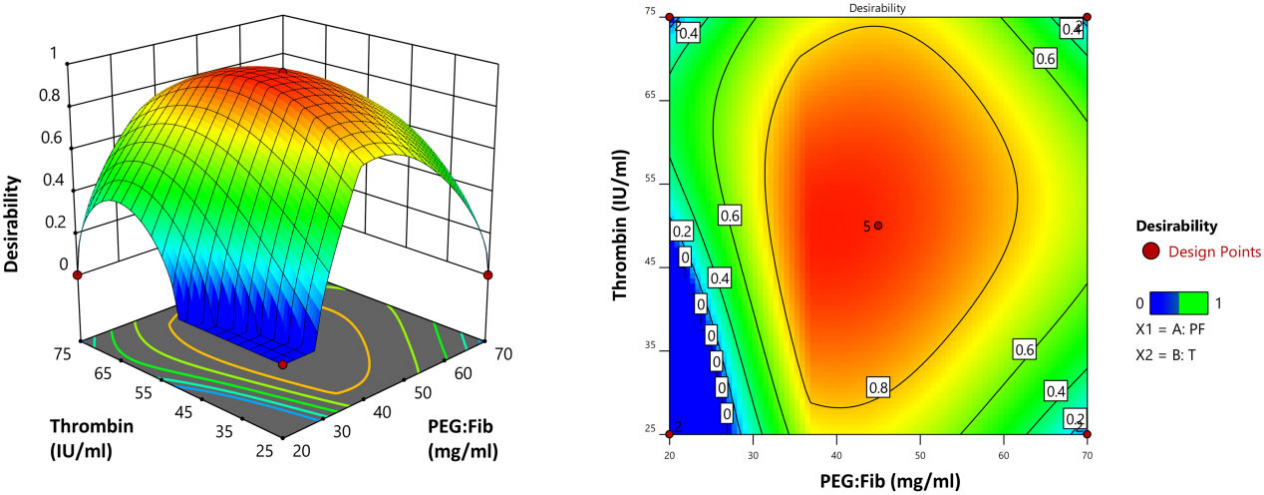
**Formulation optimization: the desirability plot based on the concentration of PEGylated fibrinogen and thrombin.** Pa, Pascal; YM, Young’s modulus; PEG, polyethylene glycol; PF, PEGylated fibrinogen; T, thrombin.

Here, we used Young’s modulus of the human ovarian cortex at reproductive age as a parameter to tailor our hydrogel, based on our hypothesis that a similar elasticity would be beneficial for the growth of isolated human preantral follicles. Hence, the optimal formulation was established by setting the specific goal of minimum deviation and targeting 3178 ± 245 Pa, which is Young’s modulus of ovarian tissue fragments in women at reproductive age (Ouni *et al.*, 2021), to obtain the optimized concentration of PEG:Fib and thrombin (Fig. 2). According to the optimization, the PEGylated fibrin hydrogel, containing 39.057 mg/ml of PEG:Fib and 50.356 IU/ml of thrombin, had the most similar Young’s modulus to the ovarian tissue fragments at reproductive age.

**Figure 3.**
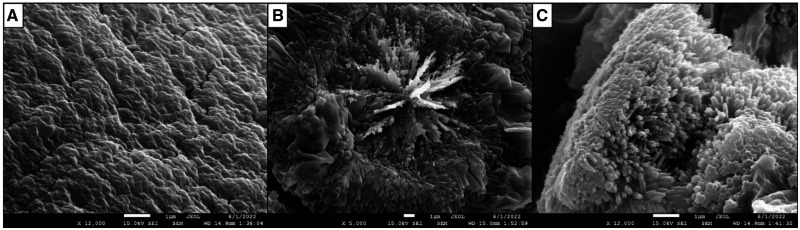
**Representative scanning electron microscopy analysis of the tailored hydrogel.** Scanning electron microscopy images of PEGylated fibrin hydrogel showing its surface (**A**), center (**B**), and filaments (**C**). Scale bars = 1 µm.

According to scanning electron microscopy analysis, our tailored hydrogels presented a homogeneous and slightly rough surface (Fig. 3). Inside, parallel bundles of long, thin filaments radiated from the center. Each filament presented a polygonal structure, predominantly with four faces, with an approximate thickness of 0.15 µm.

### Tailored PEGylated fibrin hydrogel promotes human follicle development and maintains granulosa–oocyte interaction

**Figure 4.**
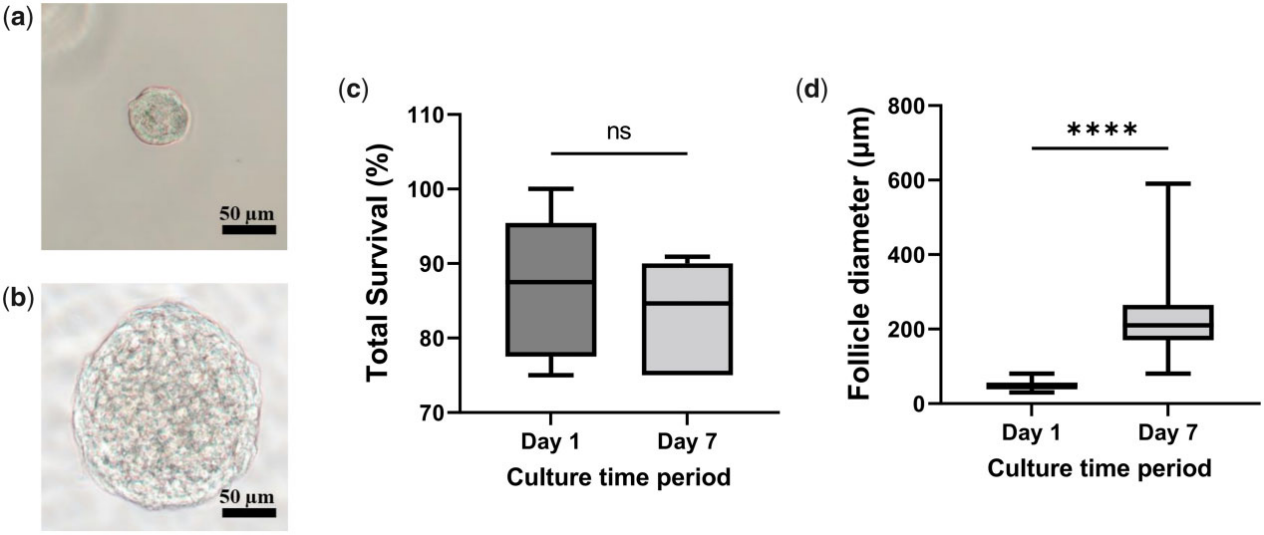
**Survival and growth of human follicles after 7 days in culture.** (**a**, **b**) Representative pictures of follicles on Days 1 and 7, respectively. (**c**) Survival and (**d**) diameter of follicles on Days 1 and 7 of *in vitro* culture. Scale bars = 50 µm. One-way ANOVA was used to statistically evaluate the data. Data in (c and d) are presented as a mean ± SD. A total of 48 follicles from five patients were used for survival analysis (c) and 146 follicles from six patients were measured: 65 on Day 1 and 81 on Day 7 (d). ns, not significant; ****P < 0.0001.

The tailored PEGylated fibrin was found to support follicle survival during the 7 days of *in vitro* culture. Indeed, the proportion of live follicles did not decrease when Day 7 (83.4 ± 6.4%) was compared to Day 1 (86.9 ± 8.9%) (Fig. 4). This hydrogel also permitted a significant growth of the isolated follicles, as evidenced by their diameter, which increased from 48.5 ± 10.8 µm on Day 1 to 222.5 ± 84.5 µm on Day 7 (Fig. 4).

**Figure 5.**
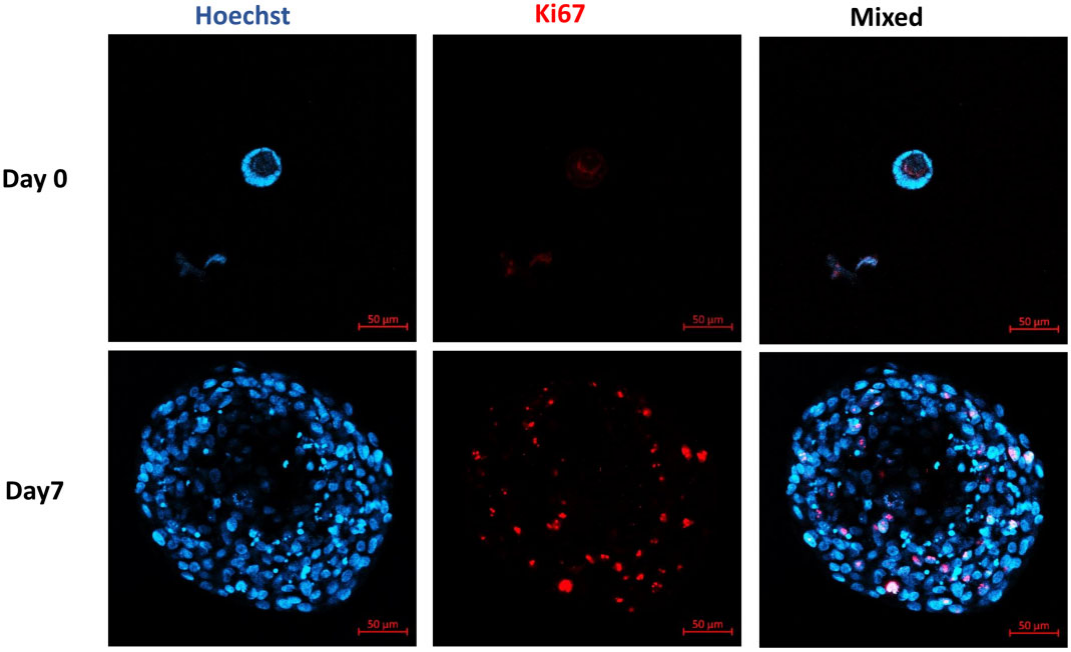
**Granulosa cell proliferation.** Representative confocal microscopy images of Ki67 expression in human follicles on Day 1 and after 7 days *in vitro*. Nuclear and Ki67-positive granulosa cells are stained blue and red, respectively. Scale bar = 50 µm.

Follicle growth in the tailored hydrogel was confirmed by Ki67 staining, which showed that all 27 follicles analyzed contained more than one Ki67-positive granulosa cell (Fig. 5). Based on the confocal pictures taken from Ki67 staining of follicles and their assessments of diameter under light microscopy, the surviving follicles were in the secondary stage of development after 7 days *in vitro* (Figs 4 and 5).

**Figure 6.**
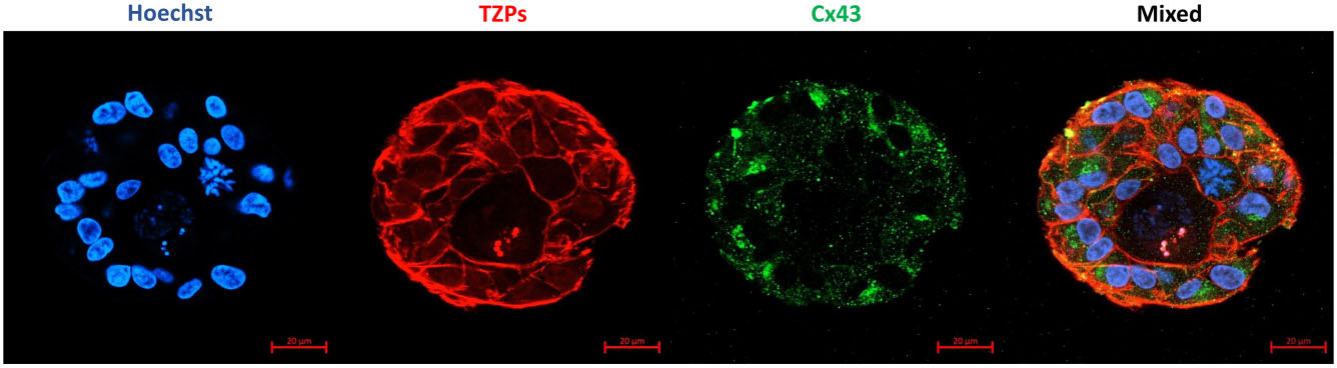
**Confocal images of localization of transzonal projections and expression of connexin 43 in human follicles after 4 days *in vitro*.** Nuclei, transzonal projections (TZPs), and connexin 43 (Cx43) are stained blue, red, and green, respectively. Scale bar = 20 µm.

Owing to the large size of the follicles on Day 7, their position in the hydrogel, and the hydrogel thickness, it was challenging to properly visualize the center of follicles, where the oocyte is localized. Hence, for evaluating cell–cell communications, including granulosa cell–oocyte, follicles were cultured for only 4 days. A total of 22 follicles were analyzed for the Cx43 and TZPs staining, and the categorization of Cx43 and TZPs was performed based on Bus *et al.* (2021). The results showed that all follicles were stained positive for Cx43. On the other hand, 45.45% of follicles had complete TZPs, while TZPs were partially and completely absent in 36.36% and 13.64% of the follicles, respectively (Fig. 6).

### Tailored PEGylated fibrin hydrogel allows cell migration

**Figure 7.**
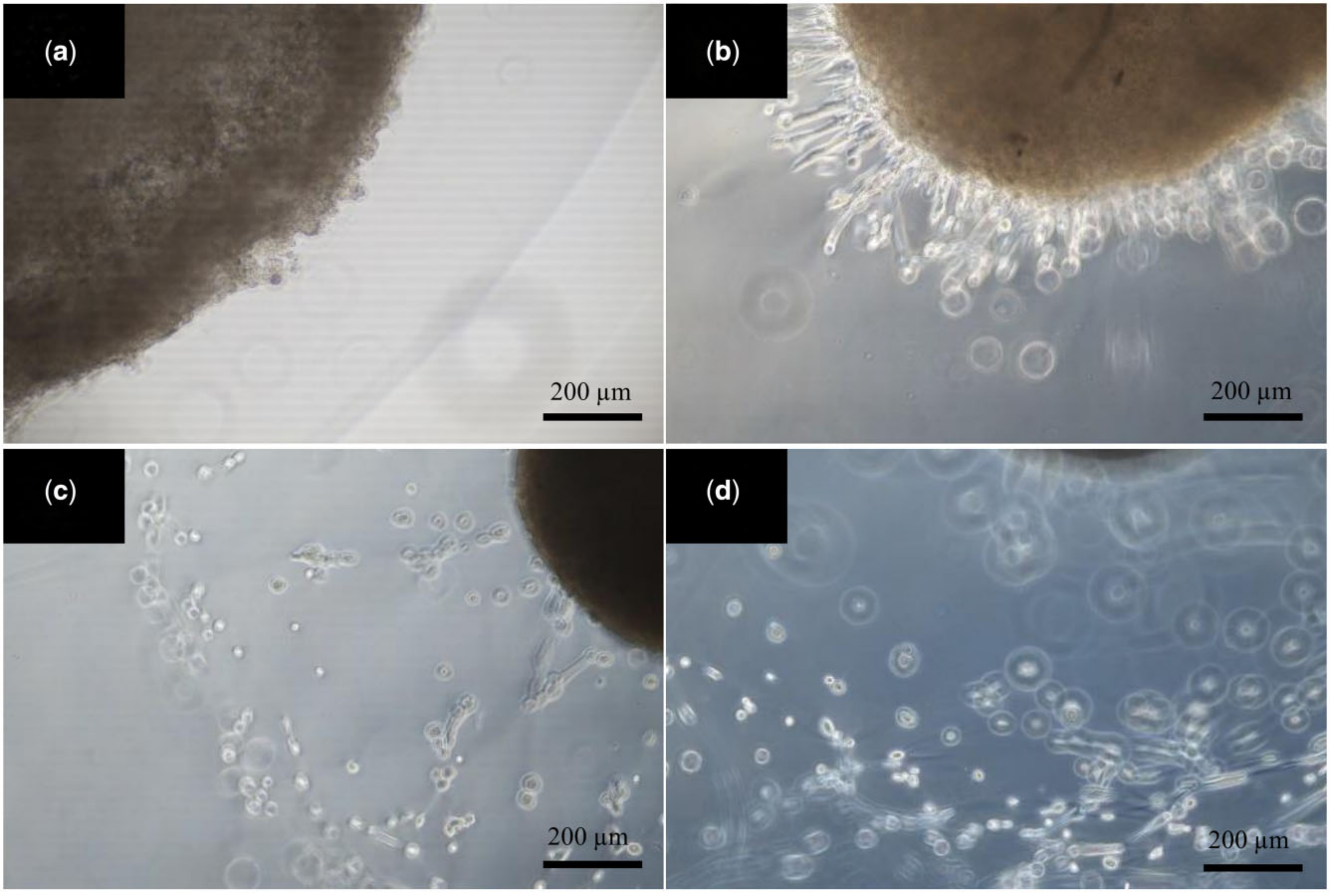
**Migration of ovarian stromal cells in the PEGylated fibrin.** Representative images of human ovarian stromal cell invasion from fibrin clot (brown) to the surrounding PEGylated fibrin hydrogel on Days 0 (**a**), 1 (**b**), 2 (**c**), and 3 (**d**) of culture, respectively. Scale bar = 200 µm.

To evaluate the ability of the PEGylated fibrin hydrogel to allow cell migration, 300 000 cells in 10 μl of fibrin clots (4 mg/ml fibrinogen, 2 IU/ml thrombin) were placed in the PEGylated fibrin hydrogels to test if the hydrogel was able to support cell migration. As pictures from Day 0 to Day 3 (Fig. 7) and the video taken on Day 4 (Video 1) demonstrate, the cells could successfully migrate from the fibrin clot to near the edge of our PEGylated fibrin hydrogel, confirming that the hydrogel could support cell migration.

## Discussion

For ovary tissue engineering, different fibrin formulations have been empirically examined to encapsulate preantral follicles. The results from the literature showed that the softer hydrogels were suitable for mouse preantral follicle encapsulation, while follicles from larger mammalian species indicated better results when encapsulated in stiffer hydrogels (West-Farrell *et al.*, 2009; Park *et al.*, 2012; Paulini *et al.*, 2016; Sadeghnia *et al.*, 2016). These reports suggest the importance of the 3D matrix’s mechanical strength for follicle development. However, the random selection of hydrogel compositions resulted in limited outcomes since the difference in the biomechanical properties between the native ovary and a 3D matrix could negatively affect the follicles owing to the influence of the mechanical strength on follicle development (West *et al.*, 2007; Smith *et al.*, 2010; Jiao and Woodruff, 2013; Brito *et al.*, 2014; Sadeghnia *et al.*, 2016). Indeed, when a follicle grows, the increase in its diameter causes the follicle to be subjected to compressive stresses from a biomaterial surrounding it. The amount of these stresses can be determined by the values of elasticity of the biomaterial and the displacements of the follicle’s size (Smith *et al.*, 2010; Dadashzadeh *et al.*, 2021). Moreover, the mechanical characteristics of the 3D matrix are critical in controlling cell–extracellular matrix interactions and dictating cell phenotype and genotype (Vedadghavami *et al.*, 2017). Therefore, it is crucial to mimic the mechanical features of the natural ovary when constructing an appropriate scaffold for follicle growth and development (Smith *et al.*, 2010; Laurent *et al.*, 2018; Kim *et al.*, 2020; Dadashzadeh *et al.*, 2021). Based on our premise that a similar elasticity would be advantageous for the growth of isolated human preantral follicles, we performed a mathematical model to mimic Young’s modulus of human ovarian cortex at reproductive age (Ouni *et al.*, 2021).

The tailored hydrogel used here supported follicle survival during the 7 days of *in vitro* culture. However, based on morphological assessment of the follicles under an inverted microscope, Chiti *et al.* (2022) reported higher viability of follicles (97.2%) compared to our results when the follicles were encapsulated in decellularized ovarian extracellular matrix hydrogel and 1% alginate (3:1 volume ratio), indicating the importance of ECM proteins for the viability of follicles. Moreover, human follicle diameter increased significantly as a result of encapsulation in the tailored hydrogel. The PEGylated fibrin hydrogel appears to be more suitable to support the development of isolated human preantral follicles than 1% alginate, which is the most used hydrogel for encapsulating human preantral follicles at present. Indeed, in 1% alginate, during the same *in vitro* culture duration, follicles grew from ∼40 to ∼60 µm in diameter (Amorim *et al.*, 2009; Camboni *et al.*, 2013; Chiti *et al.*, 2022). This result demonstrates the appropriate balance between rigidity and elasticity of our recapitulated PEGylate fibrin hydrogel to maintain the spherical follicle shape and provide a proper substrate for its growth, which resulted in a superior development of the isolated follicles.

Indeed, it has already been demonstrated that microenvironment rigidity has a crucial role in follicle fate. West *et al.* (2007) showed that matrix stiffness could affect several parameters in the encapsulated follicles, such as their rate of increasing diameter, hormone production levels, theca cell differentiation, and oocyte maturation. Moreover, this dependence of follicles on the mechanical properties of their microenvironment is species-dependent. For instance, Luyckx *et al.* (2014) demonstrated that softer fibrin hydrogels containing low fibrinogen (12.5 or 25 mg/ml) and thrombin concentrations (1 or 4 IU/ml) could support the survival of mouse follicles, yielding a recovery rate of 35% after 1 week of allografting. On the other hand, when used to encapsulate and xenograft isolated human follicles, these fibrin formulations generated a recovery rate of 2% (Amorim CA, unpublished results). These results in follicle recovery rate reveal that follicles from different species have distinctive mechanical signal requirements. This was further demonstrated in studies by Paulini *et al.* (2016) and Chiti *et al.* (2017a,b) that applied higher fibrinogen and thrombin concentrations to make a stiffer fibrin hydrogel to encapsulate and xenograft isolated human follicles. This different formulation significantly increased the follicle recovery rate to between 23% and 35% (Paulini *et al.*, 2016; Chiti *et al.*, 2017a,b). Undeniably, preserving follicle morphology and expansion can be manipulated by changing the mechanical properties of their microenvironment (Shikanov *et al.*, 2009; West *et al.*, 2007). Therefore, with the tailored hydrogel, we expected better follicle development as the follicles were provided with a microenvironment similar to a normal human ovary in terms of mechanical strength.

The Ki67 staining results confirmed the growth of follicles in the tailored PEGylated fibrin hydrogel. Studies have indicated that somatic cell proliferation and differentiation, as well as folliculogenesis, have a direct relationship with the mechanical properties of surrounding biomaterials (Xu *et al.*, 2006). Indeed, the mechanical properties of a scaffold could affect cell behavior, directing it toward proliferation, differentiation, and attachment (Baker *et al.*, 2009; Tibbitt and Anseth, 2009; Murphy *et al.*, 2012; Vedadghavami *et al.*, 2017). For instance, the speed of cell migration and proliferation can be conversely changed in stiff and soft scaffolds. Cells would proliferate faster and migrate slower in a stiffer scaffold, and *vice versa* (Ghosh *et al.*, 2007). Furthermore, Engler *et al.* (2006) reported that the elasticity of scaffolds could specify lineages of mesenchymal stem cells, in which soft, stiffer, and rigid scaffolds are neurogenic, myogenic, and osteogenic, respectively. Therefore, using a scaffold that mimics the mechanical properties of a normal tissue could induce the cells to behave in a similar fashion to when in their natural environment. In the case of follicles in the tailored hydrogel, the granulosa cells could proliferate normally, as they would in the ovarian cortex. We hypothesize that when adding isolated ovarian stromal cells to our construct, they may have a higher probability of differentiating into theca cells because of the similarity of the mechanical strength of the scaffold to the normal ovary.

Another crucial role of a biomaterial to encapsulate isolated human follicles is to maintain the gap junctions between granulosa cells and the oocyte interaction, as the development and cytoplasmic meiotic competence of the oocytes rely on these connections (Carabatsos *et al.*, 2000). Indeed, gap junctions between these cells are necessary for exchanging paracrine factors responsible for the growth of granulosa cells and the oocyte (Buccione *et al.*, 1990; Su *et al.*, 2009). Therefore, to assess the connections between granulosa cell–cell and granulosa cell–oocyte, Cx43 and TZPs staining was performed. Gap junctions are composed of Cx proteins that form connexons (hemichannels) (Meşe *et al.*, 2007). The interaction between two connexons from neighboring cells makes a functional channel. The major Cx protein for granulosa cell–cell communication is Cx43, which is crucial for follicle growth and development (Teilmann, 2005).

On the other hand, TZPs, which are essential for oocyte development, extend from cumulus cells to the oocyte for bidirectional signaling and nutrient transmission (Baena and Terasaki, 2019). TZPs are simple primary gap junctions in the early stage of follicle growth, and remodel during folliculogenesis (Clarke, 2018). They can have either an actin or tubulin backbone and lengthen during the deposition and thickening of zona pellucida (Anderson and Albertini, 1976; El-Hayek *et al.*, 2018). El-Hayek *et al.* (2018) showed that the number of TZPs had a positive correlation with oocyte diameter in antral follicles and TZPs quantification was female-age dependent.

Melton *et al.* (2001) reported that the expression of Cx43 increases in growing follicles, and Cx43 mRNA was not expressed in atretic follicles, which indicates the association of Cx43 with follicular development. Our results from Cx43 staining demonstrated that the PEGylated fibrin hydrogel provided an appropriate substrate for communications between granulosa cells, and for granulosa cell–oocyte communications. Interactions between granulosa cells and the oocyte mediate oocyte growth, its cytoplasmic development, and the exchange of essential products such as amino acids (Eppig, 1979; Pant *et al.*, 2005). Although oocytes stop growing when they physically separate from granulosa cells, they can partially grow if they block detectable gap junctions (Clarke, 2018). Moreover, Barrett *et al.* (2010) demonstrated that TZPs were damaged during cryopreservation and follicles need a recovery time after thawing to retrieve TZPs during *in vitro* culture. Trapphoff *et al.* (2010) also suggested a recovery period for restoring lost granulosa cells and oocyte connections after cryopreservation of preantral follicles. Therefore, all in all, follicles with a partial absence of TZPs can allow the partial growth of oocytes, and the TZPs may recover and regrow during the culture and recovery period of follicles. As a result, based on TZPs staining findings, we showed that 86.36% of follicles had the possibility of oocyte growth on Day 4 and only 13.64% of follicles lost their connection with oocytes and, then, the ability to support oocyte development. However, according to data from Fushii *et al.* (2021), TZPs can be reconstructed even in oocytes that lost all TZPs. These authors reported that TZPs-free denuded oocytes could have TZPs reestablished and increased in number during *in vitro* co-culture with mural granulosa cells originating from early antral follicles (Fushii *et al.*, 2021).

Besides, one of the key features of a suitable 3D matrix for tissue engineering is to support cell migration (Harley *et al.*, 2008; Werner *et al.*, 2018; Yang *et al.*, 2017). This could help with the vascularization of engineered tissue after transplantation (Lamalice *et al.*, 2007; Wu *et al.*, 2012), which is a crucial factor for cell and follicle survival. Indeed, vascular network formation is essential for exchanging hormones, growth factors, ions, and various molecules needed for folliculogenesis. Still regarding vascularization, it is also important to highlight that PEGylated fibrin has been reported to have a high angiogenesis potential (Shpichka *et al.*, 2020). While grafting of isolated human preantral follicles has not yet been tested, non-PEGylated fibrin is wholly vascularized after 7 days of grafting (Luyckx *et al.*, 2014), which appears to be faster than in human ovarian tissue after xenotransplantation (Van Eyck *et al.*, 2010). This is very promising, as it is known that reducing the post-grafting hypoxia period can increase follicle survival (Manavella *et al.*, 2018). In the case of ovary tissue engineering, our matrix could also favor the migration of ovarian stromal cells during their recruitment for further differentiation into theca cells around growing follicles (Richards *et al.*, 2018; Young and McNeilly, 2010). This is a vital aspect of the success of our approach to restoring endocrine and reproductive functions in cancer patients. Although theca cell ontogeny remains unclear, we know that their precursors are among ovarian cells in the cortex (Asiabi *et al.*, 2020). Together with granulosa cells, differentiated theca cells are responsible for the synthesis of steroid hormones in the ovarian millieu, which, in turn, play an essential role in folliculogenesis. The results from cell migration evaluation indicated the ability of PEGylated fibrin hydrogel to support migration of ovarian stromal cells. Regarding the stiffness of a scaffold that can regulate cell migration (Yin *et al.*, 2021), it appears that the mechanically tailored formulation of PEGylated fibrin hydrogel is suitable for encapsulating both human ovarian stromal cells and preantral follicles.

In conclusion, a follicle is composed of granulosa cells and an oocyte, which must be tightly connected to sustain successful oocyte development and meiotic competence. Supporting the 3D spatial structure of follicles and the cell–cell connections, as well as granulosa cell proliferation and stromal cell migration, are crucial for fabricating a scaffold for an engineered ovary. Previously, Dadashzadeh *et al.* (2021) indicated the ability of different PEGylated fibrin hydrogels to support human ovarian stromal cell survival and proliferation. In this study, the PEGylated fibrin hydrogel was shown to promote granulosa cell proliferation, follicle development, and ovarian stromal cell migration. Indeed, our results introduced an appropriate biomaterial, which resembles Young’s modulus of the cortical ovarian tissue in women of reproductive age, for encapsulating human preantral follicles. The tailored PEGylated fibrin hydrogel allowed follicles to maintain their viability and undergo radial growth. Moreover, PEGylation enhanced the fibrin stability and physical support of follicles.

## Data Availability

The data that support the findings of this study are available from the corresponding author, Christiani A. Amorim, upon reasonable request.
